# Dichlorido[tris­(benzimidazol-2-ylmeth­yl)amine]­indium(III) chloride ethanol solvate dihydrate

**DOI:** 10.1107/S1600536810029806

**Published:** 2010-07-31

**Authors:** Zuo-an Xiao, Dan Zhan

**Affiliations:** a School of Chemical Engineering and Food Science, Xiangfan University, Xiangfan 441053, People’s Republic of China

## Abstract

In the title complex, [InCl_2_(C_24_H_21_N_7_)]Cl·C_2_H_5_OH·2H_2_O, the In^III^ ion is coordinated by four N atoms from the tris­(benz­imidazol-2-ylmeth­yl)amine (NTB) ligand and two Cl atoms in a distorted octa­hedral environment. In the crystal structure, inter­molecular N—H⋯O, O—H⋯O, O—H⋯Cl and weak C—H⋯Cl hydrogen bonds connect the cations, anions and solvent mol­ecules into a three-dimensional network. The ethanol solvent mol­ecule is disordered over two sites with refined occupancies of 0.54 (2) and 0.46 (2).

## Related literature

For background information and the applications of indium complexes, see: Green *et al.* (2005 ▶); Krivokapic *et al.* (2001 ▶); Lu *et al.* (2005 ▶); Sun *et al.* (2009 ▶); Vagin *et al.* (2003 ▶). For the synthetic procedure, see: Hendriks *et al.* (1982 ▶).
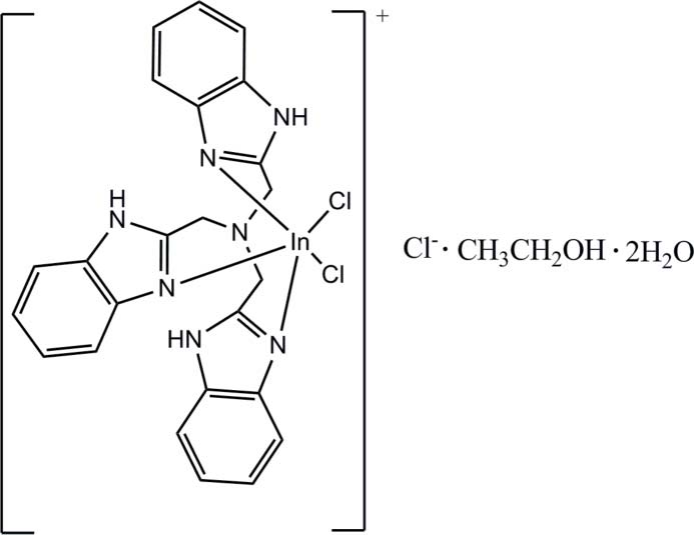


## Experimental

### 

#### Crystal data

[InCl_2_(C_24_H_21_N_7_)]Cl·C_2_H_6_O·2H_2_O
*M* *_r_* = 710.75
Monoclinic, 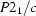

*a* = 10.4152 (10) Å
*b* = 13.7394 (13) Å
*c* = 21.903 (2) Å
β = 103.75°
*V* = 3044.4 (5) Å^3^
*Z* = 4
Mo *K*α radiation
μ = 1.08 mm^−1^
*T* = 298 K
0.26 × 0.22 × 0.20 mm

#### Data collection

Bruker SMART CCD diffractometer
Absorption correction: multi-scan (*SADABS*; Sheldrick, 1997 ▶) *T*_min_ = 0.846, *T*_max_ = 0.900
22634 measured reflections
7526 independent reflections
6941 reflections with *I* > 2σ(*I*)
*R*_int_ = 0.033

#### Refinement

*R*[*F* ^2^ > 2σ(*F* ^2^)] = 0.038
*wR*(*F* ^2^) = 0.089
*S* = 1.15
7526 reflections
403 parameters
6 restraints
H atoms treated by a mixture of independent and constrained refinement
Δρ_max_ = 0.95 e Å^−3^
Δρ_min_ = −0.55 e Å^−3^

### 

Data collection: *SMART* (Bruker, 2007 ▶); cell refinement: *SAINT-Plus* (Bruker, 2007 ▶); data reduction: *SAINT-Plus*; program(s) used to solve structure: *SHELXS97* (Sheldrick, 2008 ▶); program(s) used to refine structure: *SHELXL97* (Sheldrick, 2008 ▶); molecular graphics: *PLATON* (Spek, 2009 ▶); software used to prepare material for publication: *SHELXTL* (Sheldrick, 2008 ▶).

## Supplementary Material

Crystal structure: contains datablocks global, I. DOI: 10.1107/S1600536810029806/lh5092sup1.cif

Structure factors: contains datablocks I. DOI: 10.1107/S1600536810029806/lh5092Isup2.hkl

Additional supplementary materials:  crystallographic information; 3D view; checkCIF report

## Figures and Tables

**Table 1:** Hydrogen-bond geometry (Å, °)

*D*—H⋯*A*	*D*—H	H⋯*A*	*D*⋯*A*	*D*—H⋯*A*
C9—H9*A*⋯Cl3^i^	0.97	2.70	3.655 (3)	168
C1—H1*A*⋯Cl3^ii^	0.97	2.74	3.558 (3)	142
N7—H7*A*⋯O3^ii^	0.86	2.01	2.826 (4)	158
N5—H5*A*⋯O2^i^	0.86	1.99	2.818 (4)	161
O3—H3*B*⋯Cl3^iii^	0.83 (2)	2.35 (2)	3.144 (3)	161 (4)
O3—H3*A*⋯O2	0.82 (2)	2.05 (2)	2.861 (4)	166 (4)
N3—H3⋯O1	0.86	1.90	2.745 (12)	169
N3—H3⋯O1′	0.86	1.89	2.718 (12)	160
O2—H2*B*⋯Cl2	0.83 (2)	2.41 (2)	3.171 (3)	154 (3)
O2—H2*A*⋯Cl3	0.82 (2)	2.34 (2)	3.108 (3)	155 (3)
O1—H1⋯Cl3^iv^	0.82	2.32	3.127 (11)	167
O1′—H1′⋯Cl3^iv^	0.82	2.49	3.178 (11)	143

Symmetry codes: (i) 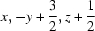; (ii) 
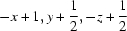
; (iii) 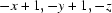; (iv) 
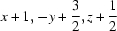
.

**Fig. 1.**
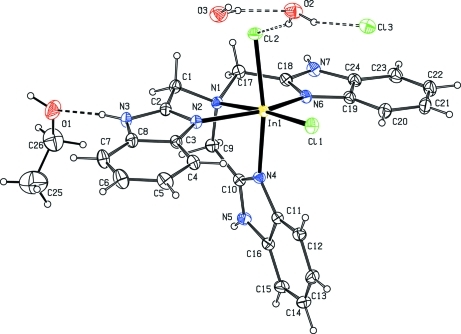
The asymmetric unit of (I), with displacement ellipsoids drawn at the 50% probability level. The disorder is not shown. Hydrogen bonds are shown as dashed lines.

**Fig. 2.**
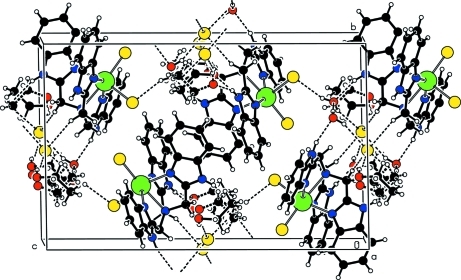
Part of the crystal structure of (I) showing hydrogen bonds as dashed lines.

## supplementary crystallographic information

### Comment

Indium complexes are widely used as radiopharmaceuticals, photoelectronic materials and catalysts (Green *et al.*, 2005; Lu *et al.*, 2005; Sun *et al.*, 2009). In the past few years, there has been considerable effort in designing Indium complexes because of their good optical properties applied in optical limiting materials (Vagin *et al.*, 2003; Krivokapic *et al.*, 2001). With this mind, the title compound, (I), was prepared and we report the crystal stucture herein.

In the molecular structure of (I), the In^III^ ion is coordinated by four N atoms from the NTB ligand and and two Cl atoms, forming a distorted octahedral coordination environmemt (Fig.1). Two benzimidazole(bzim)-N atoms (N2 and N6) occupy the axial positions, one bzim-N atom (N4), one amino N atom (N1) and two Cl atoms are located in the equatorial plane. In the crystal structure, intermolecular N—H···O, O—H···O, O—H···Cl and weak C—H···Cl hydrogen bonds form a three-dimensional network (Fig.2).

### Experimental

The NTB ligand was prepared according to literature methods (Hendriks, *et al.*, 1982). InCl_3_.4H_2_O (0.29 g,1 mmol) was added to a solution of NTB (0.41 g,1 mmol) in hot alcohol (50 ml) and refluxed for 1 h. The solution was filtered, then the filtrate was placed at room temperature and colorless single crystals suitable for an X-ray diffraction study were obtained by slow evaporation of the solvent for five days.

### Refinement

The ethanol molecule is disordered with occupancies of 0.54 (2) and 0.46 (2) for the major and minor components, respectively. H atoms bonded to C atoms were placed in idealized positions [*C*—*H*(methyl) = 0.96 Å, 0.97Å (methylene) and 0.93 Å (aromatic), with *U*_iso_(H) = 1.5*U*_eq_(methyl C) and 1.2*U*_eq_(other C). N-bound hydrogen atoms were initially located from a difference Fourier map and then placed in ideal positions with N—H = 0.86Å and *U*_iso_(H) = 1.2 *U*_eq_(N). H atoms bonded to methanol O atoms were included with O-H = 0.82Å and U_iso_(H) = 1.5U_eq_(O). H atoms bonded to water O atoms were located from difference maps and refined with distance restraints of O-H = 0.82 (1) and H···H = 1.35 (2) Å and with U_iso_(H) = 1.2U_eq_(O).

### Crystal data

**Table:**

[InCl_2_(C_24_H_21_N_7_)]Cl·C_2_H_6_O·2H_2_O	*F*(000) = 1440
*M**_r_* = 710.75	*D*_x_ = 1.551 Mg m^−^^3^
Monoclinic, *P*2_1_/*c*	Mo *K*α radiation, λ = 0.71073 Å
Hall symbol: -P 2ybc	Cell parameters from 5767 reflections
*a* = 10.4152 (10) Å	θ = 2.4–28.3°
*b* = 13.7394 (13) Å	µ = 1.08 mm^−^^1^
*c* = 21.903 (2) Å	*T* = 298 K
β = 103.75°	Block, colorless
*V* = 3044.4 (5) Å^3^	0.26 × 0.22 × 0.20 mm
*Z* = 4	

### Data collection

**Table:**

Bruker SMART CCD diffractometer	7526 independent reflections
Radiation source: fine-focus sealed tube	6941 reflections with *I* > 2σ(*I*)
graphite	*R*_int_ = 0.033
φ and ω scans	θ_max_ = 28.3°, θ_min_ = 1.9°
Absorption correction: multi-scan (*SADABS*; Sheldrick, 1997)	*h* = −13→13
*T*_min_ = 0.846, *T*_max_ = 0.900	*k* = −13→18
22634 measured reflections	*l* = −28→29

### Refinement

**Table:**

Refinement on *F*^2^	Primary atom site location: structure-invariant direct methods
Least-squares matrix: full	Secondary atom site location: difference Fourier map
*R*[*F*^2^ > 2σ(*F*^2^)] = 0.038	Hydrogen site location: inferred from neighbouring sites
*wR*(*F*^2^) = 0.089	H atoms treated by a mixture of independent and constrained refinement
*S* = 1.15	*w* = 1/[σ^2^(*F*_o_^2^) + (0.0382*P*)^2^ + 1.3452*P*] where *P* = (*F*_o_^2^ + 2*F*_c_^2^)/3
7526 reflections	(Δ/σ)_max_ < 0.001
403 parameters	Δρ_max_ = 0.95 e Å^−^^3^
6 restraints	Δρ_min_ = −0.55 e Å^−^^3^

### Special details

**Table:**

Geometry. All e.s.d.'s (except the e.s.d. in the dihedral angle between two l.s. planes) are estimated using the full covariance matrix. The cell e.s.d.'s are taken into account individually in the estimation of e.s.d.'s in distances, angles and torsion angles; correlations between e.s.d.'s in cell parameters are only used when they are defined by crystal symmetry. An approximate (isotropic) treatment of cell e.s.d.'s is used for estimating e.s.d.'s involving l.s. planes.
Refinement. Refinement of *F*^2^ against ALL reflections. The weighted *R*-factor *wR* and goodness of fit *S* are based on *F*^2^, conventional *R*-factors *R* are based on *F*, with *F* set to zero for negative *F*^2^. The threshold expression of *F*^2^ > σ(*F*^2^) is used only for calculating *R*-factors(gt) *etc*. and is not relevant to the choice of reflections for refinement. *R*-factors based on *F*^2^ are statistically about twice as large as those based on *F*, and *R*- factors based on ALL data will be even larger.

### Fractional atomic coordinates and isotropic or equivalent isotropic displacement parameters (Å^2^)

**Table:**

	*x*	*y*	*z*	*U*_iso_*/*U*_eq_	Occ. (<1)
In1	0.447613 (16)	0.710677 (12)	0.302721 (8)	0.02890 (6)	
C1	0.6729 (2)	0.85480 (19)	0.37586 (13)	0.0380 (6)	
H1A	0.7209	0.8873	0.4137	0.046*	
H1B	0.6774	0.8950	0.3400	0.046*	
C2	0.7324 (2)	0.7572 (2)	0.37027 (12)	0.0354 (5)	
C3	0.7475 (2)	0.6060 (2)	0.34430 (12)	0.0352 (5)	
C4	0.7229 (3)	0.5111 (2)	0.32325 (13)	0.0419 (6)	
H4	0.6393	0.4915	0.3011	0.050*	
C5	0.8274 (3)	0.4474 (2)	0.33659 (15)	0.0547 (8)	
H5	0.8143	0.3830	0.3234	0.066*	
C6	0.9521 (3)	0.4770 (3)	0.36927 (18)	0.0671 (10)	
H6	1.0206	0.4318	0.3769	0.081*	
C7	0.9781 (3)	0.5705 (3)	0.39078 (17)	0.0615 (9)	
H7	1.0621	0.5896	0.4127	0.074*	
C8	0.8723 (3)	0.6355 (2)	0.37807 (13)	0.0422 (6)	
C9	0.5226 (3)	0.8079 (2)	0.44197 (12)	0.0395 (6)	
H9A	0.4754	0.8572	0.4596	0.047*	
H9B	0.6107	0.8025	0.4691	0.047*	
C10	0.4529 (3)	0.71260 (19)	0.44155 (12)	0.0352 (5)	
C11	0.3720 (2)	0.5684 (2)	0.41656 (12)	0.0354 (5)	
C12	0.3344 (3)	0.4790 (2)	0.38883 (14)	0.0432 (6)	
H12	0.3404	0.4655	0.3480	0.052*	
C13	0.2875 (3)	0.4107 (2)	0.42440 (16)	0.0521 (8)	
H13	0.2617	0.3499	0.4072	0.063*	
C14	0.2782 (3)	0.4307 (3)	0.48525 (17)	0.0551 (8)	
H14	0.2449	0.3831	0.5074	0.066*	
C15	0.3163 (3)	0.5182 (3)	0.51383 (15)	0.0521 (8)	
H15	0.3101	0.5310	0.5547	0.063*	
C16	0.3648 (3)	0.5868 (2)	0.47842 (13)	0.0397 (6)	
C17	0.4499 (3)	0.92644 (19)	0.35714 (13)	0.0383 (6)	
H17A	0.4807	0.9600	0.3244	0.046*	
H17B	0.4554	0.9710	0.3920	0.046*	
C18	0.3108 (3)	0.8943 (2)	0.33274 (12)	0.0363 (5)	
C19	0.1407 (3)	0.8058 (2)	0.28851 (12)	0.0366 (6)	
C20	0.0541 (3)	0.7343 (2)	0.25857 (15)	0.0478 (7)	
H20	0.0843	0.6735	0.2495	0.057*	
C21	−0.0787 (3)	0.7572 (3)	0.24282 (16)	0.0566 (8)	
H21	−0.1390	0.7109	0.2225	0.068*	
C22	−0.1246 (3)	0.8477 (3)	0.25658 (16)	0.0566 (9)	
H22	−0.2148	0.8604	0.2448	0.068*	
C23	−0.0411 (3)	0.9185 (3)	0.28691 (15)	0.0526 (8)	
H23	−0.0726	0.9784	0.2967	0.063*	
C24	0.0937 (3)	0.8965 (2)	0.30258 (13)	0.0408 (6)	
C25	1.061 (3)	0.7795 (16)	0.5779 (14)	0.163 (9)	0.54 (2)
H25A	1.0751	0.7203	0.5572	0.244*	0.54 (2)
H25B	1.0014	0.7672	0.6045	0.244*	0.54 (2)
H25C	1.1435	0.8028	0.6029	0.244*	0.54 (2)
C26	1.0026 (12)	0.8538 (10)	0.5302 (6)	0.101 (5)	0.54 (2)
H26A	0.9071	0.8488	0.5224	0.121*	0.54 (2)
H26B	1.0271	0.9174	0.5485	0.121*	0.54 (2)
C25'	1.058 (3)	0.8492 (17)	0.5828 (15)	0.140 (9)	0.46 (2)
H25D	1.1351	0.8561	0.6166	0.209*	0.46 (2)
H25E	0.9843	0.8298	0.5993	0.209*	0.46 (2)
H25F	1.0383	0.9104	0.5613	0.209*	0.46 (2)
C26'	1.0832 (19)	0.7717 (14)	0.5365 (8)	0.115 (7)	0.46 (2)
H26C	1.1710	0.7449	0.5522	0.138*	0.46 (2)
H26D	1.0201	0.7192	0.5346	0.138*	0.46 (2)
Cl1	0.35306 (7)	0.57777 (5)	0.23656 (3)	0.04797 (17)	
Cl2	0.49870 (7)	0.81165 (5)	0.21877 (3)	0.04437 (16)	
Cl3	0.30232 (9)	0.53452 (6)	0.00493 (4)	0.0574 (2)	
N1	0.53335 (19)	0.83983 (15)	0.37826 (9)	0.0316 (4)	
N2	0.6621 (2)	0.68466 (16)	0.34018 (10)	0.0341 (5)	
N3	0.8591 (2)	0.73183 (19)	0.39420 (12)	0.0431 (6)	
H3	0.9204	0.7686	0.4155	0.052*	
N4	0.4266 (2)	0.65021 (16)	0.39445 (10)	0.0339 (5)	
N5	0.4164 (2)	0.67908 (18)	0.49227 (11)	0.0412 (5)	
H5A	0.4238	0.7095	0.5272	0.049*	
N6	0.2781 (2)	0.80711 (16)	0.30802 (10)	0.0350 (5)	
N7	0.2042 (2)	0.95009 (17)	0.33077 (11)	0.0421 (5)	
H7A	0.2045	1.0087	0.3446	0.050*	
O2	0.4684 (3)	0.6888 (2)	0.09360 (12)	0.0655 (7)	
H2A	0.416 (3)	0.644 (2)	0.0807 (16)	0.079*	
H2B	0.460 (4)	0.706 (3)	0.1286 (11)	0.079*	
O3	0.7331 (3)	0.6250 (2)	0.10069 (13)	0.0683 (7)	
H3A	0.662 (3)	0.653 (3)	0.0997 (17)	0.082*	
H3B	0.726 (4)	0.595 (3)	0.0673 (13)	0.082*	
O1	1.0355 (12)	0.8506 (10)	0.4735 (6)	0.079 (3)	0.54 (2)
H1	1.1026	0.8828	0.4753	0.118*	0.54 (2)
O1'	1.0731 (18)	0.8074 (14)	0.4765 (8)	0.097 (5)	0.46 (2)
H1'	1.0988	0.8639	0.4787	0.146*	0.46 (2)

### Atomic displacement parameters (Å^2^)

**Table:**

	*U*^11^	*U*^22^	*U*^33^	*U*^12^	*U*^13^	*U*^23^
In1	0.02836 (9)	0.02947 (10)	0.02775 (10)	0.00063 (6)	0.00442 (6)	−0.00344 (6)
C1	0.0342 (13)	0.0353 (14)	0.0431 (15)	−0.0046 (10)	0.0066 (11)	−0.0048 (11)
C2	0.0305 (12)	0.0396 (14)	0.0356 (13)	−0.0011 (10)	0.0065 (10)	−0.0018 (11)
C3	0.0306 (12)	0.0425 (15)	0.0323 (12)	0.0054 (10)	0.0071 (10)	−0.0006 (11)
C4	0.0411 (14)	0.0450 (16)	0.0378 (14)	0.0049 (12)	0.0054 (11)	−0.0067 (12)
C5	0.0592 (19)	0.0491 (19)	0.0544 (19)	0.0177 (15)	0.0111 (15)	−0.0086 (15)
C6	0.0522 (19)	0.066 (2)	0.079 (3)	0.0278 (17)	0.0071 (18)	−0.0071 (19)
C7	0.0341 (15)	0.074 (2)	0.070 (2)	0.0131 (15)	0.0005 (15)	−0.0025 (19)
C8	0.0337 (13)	0.0485 (17)	0.0437 (15)	0.0030 (12)	0.0080 (11)	−0.0016 (13)
C9	0.0470 (15)	0.0390 (15)	0.0315 (13)	−0.0032 (12)	0.0076 (11)	−0.0066 (11)
C10	0.0343 (12)	0.0371 (14)	0.0329 (13)	0.0027 (10)	0.0052 (10)	0.0017 (10)
C11	0.0311 (12)	0.0368 (14)	0.0372 (13)	0.0009 (10)	0.0058 (10)	0.0053 (11)
C12	0.0445 (15)	0.0382 (15)	0.0450 (16)	−0.0008 (12)	0.0066 (12)	0.0024 (12)
C13	0.0454 (16)	0.0393 (17)	0.068 (2)	−0.0067 (13)	0.0066 (15)	0.0069 (15)
C14	0.0465 (17)	0.053 (2)	0.065 (2)	−0.0027 (14)	0.0120 (15)	0.0229 (16)
C15	0.0501 (17)	0.064 (2)	0.0441 (16)	0.0020 (15)	0.0152 (14)	0.0172 (15)
C16	0.0370 (13)	0.0422 (15)	0.0389 (14)	0.0023 (11)	0.0067 (11)	0.0070 (12)
C17	0.0402 (14)	0.0308 (13)	0.0423 (15)	0.0016 (11)	0.0066 (11)	−0.0046 (11)
C18	0.0373 (13)	0.0352 (14)	0.0353 (13)	0.0056 (10)	0.0066 (10)	−0.0017 (11)
C19	0.0321 (12)	0.0443 (16)	0.0340 (13)	0.0044 (11)	0.0088 (10)	0.0032 (11)
C20	0.0416 (15)	0.0513 (18)	0.0489 (17)	−0.0063 (13)	0.0074 (13)	−0.0049 (14)
C21	0.0365 (15)	0.078 (2)	0.0540 (19)	−0.0108 (15)	0.0074 (14)	−0.0010 (17)
C22	0.0332 (14)	0.081 (3)	0.0549 (19)	0.0072 (15)	0.0090 (13)	0.0102 (17)
C23	0.0444 (16)	0.062 (2)	0.0530 (18)	0.0169 (14)	0.0144 (14)	0.0100 (15)
C24	0.0380 (14)	0.0466 (16)	0.0379 (14)	0.0071 (12)	0.0095 (11)	0.0036 (12)
C25	0.186 (19)	0.160 (19)	0.153 (19)	0.005 (18)	0.063 (15)	0.027 (19)
C26	0.097 (7)	0.124 (10)	0.081 (9)	0.009 (7)	0.022 (6)	−0.021 (6)
C25'	0.132 (14)	0.16 (2)	0.14 (2)	−0.010 (17)	0.058 (13)	0.017 (18)
C26'	0.114 (12)	0.120 (14)	0.102 (12)	−0.042 (10)	0.010 (9)	0.017 (10)
Cl1	0.0516 (4)	0.0418 (4)	0.0476 (4)	−0.0079 (3)	0.0061 (3)	−0.0161 (3)
Cl2	0.0537 (4)	0.0435 (4)	0.0372 (3)	−0.0036 (3)	0.0134 (3)	0.0040 (3)
Cl3	0.0627 (5)	0.0534 (5)	0.0556 (5)	0.0060 (4)	0.0133 (4)	0.0069 (4)
N1	0.0316 (10)	0.0297 (11)	0.0327 (11)	−0.0017 (8)	0.0060 (8)	−0.0022 (8)
N2	0.0304 (10)	0.0347 (11)	0.0360 (11)	0.0009 (8)	0.0053 (9)	−0.0045 (9)
N3	0.0291 (11)	0.0495 (15)	0.0471 (14)	−0.0048 (10)	0.0015 (10)	−0.0036 (11)
N4	0.0365 (11)	0.0338 (12)	0.0309 (11)	−0.0014 (9)	0.0071 (9)	0.0001 (9)
N5	0.0473 (13)	0.0463 (14)	0.0311 (11)	0.0003 (11)	0.0113 (10)	−0.0002 (10)
N6	0.0323 (10)	0.0351 (12)	0.0365 (11)	0.0030 (9)	0.0057 (9)	−0.0032 (9)
N7	0.0437 (12)	0.0378 (13)	0.0440 (13)	0.0089 (10)	0.0090 (10)	−0.0048 (10)
O2	0.087 (2)	0.0677 (18)	0.0421 (13)	−0.0024 (13)	0.0163 (13)	−0.0034 (12)
O3	0.0772 (17)	0.0528 (16)	0.0736 (18)	−0.0008 (13)	0.0156 (15)	0.0047 (13)
O1	0.073 (5)	0.092 (8)	0.068 (5)	−0.036 (5)	0.010 (4)	−0.017 (5)
O1'	0.092 (9)	0.094 (10)	0.087 (6)	−0.042 (7)	−0.015 (6)	0.005 (8)

### Geometric parameters (Å, °)

**Table:**

In1—N2	2.218 (2)	C17—N1	1.481 (3)
In1—N4	2.232 (2)	C17—C18	1.487 (4)
In1—N6	2.233 (2)	C17—H17A	0.9700
In1—Cl1	2.3928 (7)	C17—H17B	0.9700
In1—N1	2.446 (2)	C18—N6	1.326 (3)
In1—Cl2	2.4604 (7)	C18—N7	1.341 (3)
C1—N1	1.481 (3)	C19—C20	1.389 (4)
C1—C2	1.495 (4)	C19—N6	1.392 (3)
C1—H1A	0.9700	C19—C24	1.399 (4)
C1—H1B	0.9700	C20—C21	1.380 (4)
C2—N2	1.317 (3)	C20—H20	0.9300
C2—N3	1.344 (3)	C21—C22	1.391 (5)
C3—C4	1.386 (4)	C21—H21	0.9300
C3—N2	1.390 (3)	C22—C23	1.367 (5)
C3—C8	1.394 (4)	C22—H22	0.9300
C4—C5	1.374 (4)	C23—C24	1.397 (4)
C4—H4	0.9300	C23—H23	0.9300
C5—C6	1.386 (5)	C24—N7	1.383 (4)
C5—H5	0.9300	C25—C26	1.48 (3)
C6—C7	1.374 (5)	C25—H25A	0.9600
C6—H6	0.9300	C25—H25B	0.9600
C7—C8	1.393 (4)	C25—H25C	0.9600
C7—H7	0.9300	C26—O1	1.366 (17)
C8—N3	1.386 (4)	C26—H26A	0.9700
C9—N1	1.492 (3)	C26—H26B	0.9700
C9—C10	1.495 (4)	C25'—C26'	1.54 (4)
C9—H9A	0.9700	C25'—H25D	0.9600
C9—H9B	0.9700	C25'—H25E	0.9600
C10—N4	1.319 (3)	C25'—H25F	0.9600
C10—N5	1.338 (3)	C26'—O1'	1.38 (2)
C11—C12	1.385 (4)	C26'—H26C	0.9700
C11—N4	1.397 (3)	C26'—H26D	0.9700
C11—C16	1.398 (4)	N3—H3	0.8600
C12—C13	1.380 (4)	N5—H5A	0.8600
C12—H12	0.9300	N7—H7A	0.8600
C13—C14	1.387 (5)	O2—H2A	0.823 (17)
C13—H13	0.9300	O2—H2B	0.825 (18)
C14—C15	1.369 (5)	O3—H3A	0.823 (18)
C14—H14	0.9300	O3—H3B	0.829 (18)
C15—C16	1.390 (4)	O1—H1	0.8200
C15—H15	0.9300	O1'—H1'	0.8195
C16—N5	1.382 (4)		
			
N2—In1—N4	84.98 (8)	C21—C20—C19	117.1 (3)
N2—In1—N6	144.67 (8)	C21—C20—H20	121.4
N4—In1—N6	85.77 (8)	C19—C20—H20	121.4
N2—In1—Cl1	109.53 (6)	C20—C21—C22	121.7 (3)
N4—In1—Cl1	98.44 (6)	C20—C21—H21	119.1
N6—In1—Cl1	105.52 (6)	C22—C21—H21	119.1
N2—In1—N1	72.12 (7)	C23—C22—C21	122.0 (3)
N4—In1—N1	76.07 (7)	C23—C22—H22	119.0
N6—In1—N1	72.56 (7)	C21—C22—H22	119.0
Cl1—In1—N1	174.22 (5)	C22—C23—C24	116.8 (3)
N2—In1—Cl2	89.41 (6)	C22—C23—H23	121.6
N4—In1—Cl2	165.30 (6)	C24—C23—H23	121.6
N6—In1—Cl2	91.06 (6)	N7—C24—C23	132.5 (3)
Cl1—In1—Cl2	96.24 (3)	N7—C24—C19	105.9 (2)
N1—In1—Cl2	89.28 (5)	C23—C24—C19	121.5 (3)
N1—C1—C2	107.9 (2)	O1—C26—C25	118.0 (15)
N1—C1—H1A	110.1	O1—C26—H26A	107.8
C2—C1—H1A	110.1	C25—C26—H26A	107.8
N1—C1—H1B	110.1	O1—C26—H26B	107.8
C2—C1—H1B	110.1	C25—C26—H26B	107.8
H1A—C1—H1B	108.4	H26A—C26—H26B	107.1
N2—C2—N3	112.1 (2)	C26'—C25'—H25D	109.5
N2—C2—C1	121.8 (2)	C26'—C25'—H25E	109.5
N3—C2—C1	126.0 (2)	H25D—C25'—H25E	109.5
C4—C3—N2	130.2 (2)	C26'—C25'—H25F	109.5
C4—C3—C8	121.7 (2)	H25D—C25'—H25F	109.5
N2—C3—C8	108.1 (2)	H25E—C25'—H25F	109.5
C5—C4—C3	117.0 (3)	O1'—C26'—C25'	113.3 (18)
C5—C4—H4	121.5	O1'—C26'—H26C	108.9
C3—C4—H4	121.5	C25'—C26'—H26C	108.9
C4—C5—C6	121.4 (3)	O1'—C26'—H26D	108.9
C4—C5—H5	119.3	C25'—C26'—H26D	108.9
C6—C5—H5	119.3	H26C—C26'—H26D	107.7
C7—C6—C5	122.4 (3)	In1—Cl1—O2	91.52 (5)
C7—C6—H6	118.8	C17—N1—C1	112.7 (2)
C5—C6—H6	118.8	C17—N1—C9	111.1 (2)
C6—C7—C8	116.7 (3)	C1—N1—C9	111.5 (2)
C6—C7—H7	121.7	C17—N1—In1	106.19 (14)
C8—C7—H7	121.7	C1—N1—In1	106.27 (15)
N3—C8—C7	133.1 (3)	C9—N1—In1	108.81 (15)
N3—C8—C3	106.0 (2)	C2—N2—C3	106.5 (2)
C7—C8—C3	120.9 (3)	C2—N2—In1	117.06 (17)
N1—C9—C10	113.3 (2)	C3—N2—In1	136.28 (17)
N1—C9—H9A	108.9	C2—N3—C8	107.2 (2)
C10—C9—H9A	108.9	C2—N3—O1	122.7 (4)
N1—C9—H9B	108.9	C8—N3—O1	129.6 (4)
C10—C9—H9B	108.9	C2—N3—H3	126.4
H9A—C9—H9B	107.7	C8—N3—H3	126.4
N4—C10—N5	112.2 (2)	C10—N4—C11	106.1 (2)
N4—C10—C9	125.7 (2)	C10—N4—In1	114.55 (17)
N5—C10—C9	121.9 (2)	C11—N4—In1	138.28 (17)
C12—C11—N4	131.1 (3)	C10—N5—C16	107.8 (2)
C12—C11—C16	120.7 (3)	C10—N5—H5A	126.1
N4—C11—C16	108.1 (2)	C16—N5—H5A	126.1
C13—C12—C11	117.1 (3)	C18—N6—C19	106.4 (2)
C13—C12—H12	121.4	C18—N6—In1	115.33 (17)
C11—C12—H12	121.4	C19—N6—In1	138.08 (18)
C12—C13—C14	121.5 (3)	C18—N7—C24	107.8 (2)
C12—C13—H13	119.3	C18—N7—H7A	126.1
C14—C13—H13	119.3	C24—N7—H7A	126.1
C15—C14—C13	122.3 (3)	O3—O2—Cl3	102.18 (10)
C15—C14—H14	118.8	O3—O2—Cl1	108.16 (10)
C13—C14—H14	118.8	Cl3—O2—Cl1	90.17 (7)
C14—C15—C16	116.4 (3)	O3—O2—H2A	110 (3)
C14—C15—H15	121.8	Cl1—O2—H2A	71 (3)
C16—C15—H15	121.8	O3—O2—H2B	110 (3)
N5—C16—C15	132.4 (3)	Cl3—O2—H2B	128 (3)
N5—C16—C11	105.7 (2)	H2A—O2—H2B	109 (3)
C15—C16—C11	121.9 (3)	O2—O3—H3B	103 (3)
N1—C17—C18	108.9 (2)	H3A—O3—H3B	108 (3)
N1—C17—H17A	109.9	C26—O1—N3	108.6 (8)
C18—C17—H17A	109.9	C26—O1—H1	109.4
N1—C17—H17B	109.9	N3—O1—H1	141.9
C18—C17—H17B	109.9	C26—O1—H1'	106.6
H17A—C17—H17B	108.3	N3—O1—H1'	139.3
N6—C18—N7	111.9 (2)	C26'—O1'—H3	115.6
N6—C18—C17	123.3 (2)	C26'—O1'—H1	113.8
N7—C18—C17	124.8 (2)	H3—O1'—H1	116.6
C20—C19—N6	131.2 (3)	C26'—O1'—H1'	109.2
C20—C19—C24	120.8 (3)	H3—O1'—H1'	121.1
N6—C19—C24	108.0 (2)		
			
N1—C1—C2—N2	32.2 (3)	C4—C3—N2—In1	1.4 (5)
N1—C1—C2—N3	−147.5 (3)	C8—C3—N2—In1	−175.54 (19)
N2—C3—C4—C5	−177.2 (3)	N4—In1—N2—C2	−92.1 (2)
C8—C3—C4—C5	−0.6 (4)	N6—In1—N2—C2	−16.7 (3)
C3—C4—C5—C6	−0.4 (5)	Cl1—In1—N2—C2	170.69 (18)
C4—C5—C6—C7	0.8 (6)	N1—In1—N2—C2	−15.16 (18)
C5—C6—C7—C8	−0.1 (6)	Cl2—In1—N2—C2	74.28 (19)
C6—C7—C8—N3	176.6 (3)	N4—In1—N2—C3	82.8 (3)
C6—C7—C8—C3	−1.0 (5)	N6—In1—N2—C3	158.2 (2)
C4—C3—C8—N3	−176.8 (3)	Cl1—In1—N2—C3	−14.4 (3)
N2—C3—C8—N3	0.5 (3)	N1—In1—N2—C3	159.8 (3)
C4—C3—C8—C7	1.4 (4)	Cl2—In1—N2—C3	−110.8 (2)
N2—C3—C8—C7	178.7 (3)	N2—C2—N3—C8	0.4 (3)
N1—C9—C10—N4	13.9 (4)	C1—C2—N3—C8	−179.8 (3)
N1—C9—C10—N5	−169.9 (2)	N2—C2—N3—O1	−171.9 (4)
N4—C11—C12—C13	177.5 (3)	C1—C2—N3—O1	7.9 (5)
C16—C11—C12—C13	1.3 (4)	C7—C8—N3—C2	−178.4 (3)
C11—C12—C13—C14	0.1 (4)	C3—C8—N3—C2	−0.5 (3)
C12—C13—C14—C15	−0.9 (5)	C7—C8—N3—O1	−6.8 (6)
C13—C14—C15—C16	0.3 (5)	C3—C8—N3—O1	171.0 (4)
C14—C15—C16—N5	−176.8 (3)	N5—C10—N4—C11	−1.7 (3)
C14—C15—C16—C11	1.2 (4)	C9—C10—N4—C11	174.7 (2)
C12—C11—C16—N5	176.5 (2)	N5—C10—N4—In1	168.72 (17)
N4—C11—C16—N5	−0.5 (3)	C9—C10—N4—In1	−14.8 (3)
C12—C11—C16—C15	−2.0 (4)	C12—C11—N4—C10	−175.2 (3)
N4—C11—C16—C15	−179.0 (2)	C16—C11—N4—C10	1.4 (3)
N1—C17—C18—N6	−27.6 (4)	C12—C11—N4—In1	17.9 (4)
N1—C17—C18—N7	155.0 (3)	C16—C11—N4—In1	−165.52 (19)
N6—C19—C20—C21	−177.2 (3)	N2—In1—N4—C10	80.35 (18)
C24—C19—C20—C21	0.9 (4)	N6—In1—N4—C10	−65.52 (18)
C19—C20—C21—C22	−0.4 (5)	Cl1—In1—N4—C10	−170.61 (17)
C20—C21—C22—C23	−0.6 (5)	N1—In1—N4—C10	7.55 (17)
C21—C22—C23—C24	1.2 (5)	Cl2—In1—N4—C10	12.4 (4)
C22—C23—C24—N7	177.5 (3)	N2—In1—N4—C11	−113.5 (3)
C22—C23—C24—C19	−0.7 (4)	N6—In1—N4—C11	100.6 (3)
C20—C19—C24—N7	−179.0 (3)	Cl1—In1—N4—C11	−4.5 (3)
N6—C19—C24—N7	−0.4 (3)	N1—In1—N4—C11	173.7 (3)
C20—C19—C24—C23	−0.3 (4)	Cl2—In1—N4—C11	178.55 (17)
N6—C19—C24—C23	178.2 (3)	N4—C10—N5—C16	1.4 (3)
N2—In1—Cl1—O2	−82.46 (8)	C9—C10—N5—C16	−175.2 (2)
N4—In1—Cl1—O2	−170.10 (7)	C15—C16—N5—C10	177.8 (3)
N6—In1—Cl1—O2	101.98 (8)	C11—C16—N5—C10	−0.5 (3)
Cl2—In1—Cl1—O2	9.13 (5)	N7—C18—N6—C19	0.1 (3)
C18—C17—N1—C1	153.9 (2)	C17—C18—N6—C19	−177.7 (2)
C18—C17—N1—C9	−80.2 (3)	N7—C18—N6—In1	176.14 (17)
C18—C17—N1—In1	38.0 (2)	C17—C18—N6—In1	−1.6 (3)
C2—C1—N1—C17	−155.8 (2)	C20—C19—N6—C18	178.5 (3)
C2—C1—N1—C9	78.6 (3)	C24—C19—N6—C18	0.2 (3)
C2—C1—N1—In1	−39.9 (2)	C20—C19—N6—In1	3.9 (5)
C10—C9—N1—C17	111.3 (2)	C24—C19—N6—In1	−174.4 (2)
C10—C9—N1—C1	−122.2 (2)	N2—In1—N6—C18	19.4 (3)
C10—C9—N1—In1	−5.3 (3)	N4—In1—N6—C18	94.52 (19)
N2—In1—N1—C17	150.47 (17)	Cl1—In1—N6—C18	−167.88 (18)
N4—In1—N1—C17	−120.38 (16)	N1—In1—N6—C18	17.80 (18)
N6—In1—N1—C17	−30.48 (15)	Cl2—In1—N6—C18	−71.11 (19)
Cl2—In1—N1—C17	60.86 (15)	N2—In1—N6—C19	−166.3 (2)
N2—In1—N1—C1	30.28 (16)	N4—In1—N6—C19	−91.1 (3)
N4—In1—N1—C1	119.44 (16)	Cl1—In1—N6—C19	6.5 (3)
N6—In1—N1—C1	−150.67 (17)	N1—In1—N6—C19	−167.8 (3)
Cl2—In1—N1—C1	−59.33 (15)	Cl2—In1—N6—C19	103.2 (3)
N2—In1—N1—C9	−89.91 (17)	N6—C18—N7—C24	−0.4 (3)
N4—In1—N1—C9	−0.75 (16)	C17—C18—N7—C24	177.3 (3)
N6—In1—N1—C9	89.14 (17)	C23—C24—N7—C18	−177.9 (3)
Cl2—In1—N1—C9	−179.52 (16)	C19—C24—N7—C18	0.5 (3)
N3—C2—N2—C3	−0.1 (3)	In1—Cl1—O2—O3	86.39 (9)
C1—C2—N2—C3	−179.9 (2)	In1—Cl1—O2—Cl3	−170.73 (6)
N3—C2—N2—In1	176.25 (17)	C25—C26—O1—N3	89.1 (15)
C1—C2—N2—In1	−3.6 (3)	C2—N3—O1—C26	70.9 (9)
C4—C3—N2—C2	176.7 (3)	C8—N3—O1—C26	−99.5 (8)
C8—C3—N2—C2	−0.3 (3)	C25'—C26'—O1'—H3	−107.0

### Hydrogen-bond geometry (Å, °)

**Table:**

*D*—H···*A*	*D*—H	H···*A*	*D*···*A*	*D*—H···*A*
C9—H9A···Cl3^i^	0.97	2.70	3.655 (3)	168
C1—H1A···Cl3^ii^	0.97	2.74	3.558 (3)	142
N7—H7A···O3^ii^	0.86	2.01	2.826 (4)	158
N5—H5A···O2^i^	0.86	1.99	2.818 (4)	161
O3—H3B···Cl3^iii^	0.83 (2)	2.35 (2)	3.144 (3)	161 (4)
O3—H3A···O2	0.82 (2)	2.05 (2)	2.861 (4)	166 (4)
N3—H3···O1	0.86	1.90	2.745 (12)	169
N3—H3···O1'	0.86	1.89	2.718 (12)	160
O2—H2B···Cl2	0.83 (2)	2.41 (2)	3.171 (3)	154 (3)
O2—H2A···Cl3	0.82 (2)	2.34 (2)	3.108 (3)	155 (3)
O1—H1···Cl3^iv^	0.82	2.32	3.127 (11)	167
O1'—H1'···Cl3^iv^	0.82	2.49	3.178 (11)	143

Symmetry codes: (i) *x*, −*y*+3/2, *z*+1/2; (ii) −*x*+1, *y*+1/2, −*z*+1/2; (iii) −*x*+1, −*y*+1, −*z*; (iv) *x*+1, −*y*+3/2, *z*+1/2.
